# Successful management of immunotherapy-resistant respiratory failure in anti-mitochondrial M2 antibody-positive myositis by modified lung volume recruitment therapy: A case report

**DOI:** 10.1097/MD.0000000000040912

**Published:** 2024-12-13

**Authors:** Seiya Takahashi, Hiroyasu Inoue, Shizuki Amano, Takahiro Shinohara, Sara Mori, Kaho Onizawa, Yoko Nabeshima, Hiroyasu Komuro, Taro Yasumoto, Rihito Mitsuhashi, Daishi Watanabe, Kazuki Komaba, Akinori Futamura, Satoshi Nogawa, Ryuta Kinno

**Affiliations:** a Department of Neurology, Showa University Fujigaoka Hospital, Yokohama, Kanagawa, Japan; b Department of Rehabilitation, Showa University Fujigaoka Hospital, Yokohama, Kanagawa, Japan; c Department of Clinical Engineering, Showa University Fujigaoka Hospital, Yokohama, Kanagawa, Japan.

**Keywords:** autoimmune myositis, lung volume recruitment, respiratory failure, respiratory muscle weakness

## Abstract

**Rationale:**

Anti-mitochondrial antibodies (AMA) M2-positive myositis can lead to severe respiratory failure. Traditional immunotherapies sometimes fail to address respiratory failure. Herein, this CARE-compliant case report described a patient with AMA-M2-positive myositis who recovered from ventilation with tracheostomy owing to immunotherapy-resistant respiratory failure to spontaneous breathing after modified lung volume recruitment (mLVR) therapy.

**Patient concerns:**

A 60-year-old man experienced general fatigue and shortness of breath. The patient had a notable degree of proximal muscle weakness. Blood test results revealed hyperCKemia. The serum AMA-M2 antibody was positive. A muscle magnetic resonance imaging revealed diffuse abnormal hyperintensities in both lower limb muscles. Needle electromyography demonstrated fibrillation and positive sharp waves at rest with early recruitment, suggesting myogenic changes. Progressive muscle weakness and clinical findings fulfilled the criteria for definite idiopathic inflammatory myopathy. We diagnosed him with AMA-M2-positive myositis. Notably, his thoracic mobility was decreased, resulting in CO2 narcosis, requiring ventilation. Two courses of intravenous methylprednisolone for 3 days followed by oral prednisolone for myopathy. However, his respiratory function remained compromised, resulting in a tracheostomy.

**Diagnoses:**

We diagnosed him with severe immunotherapy-resistant respiratory failure due to AMA-positive myositis that required ventilation and tracheostomy.

**Interventions:**

To address respiratory failure, this patient underwent the mLVR therapy using the LIC TRAINER (LT). The pressure was adjusted by the operator using a bag-valve mask (BVM), starting at 1500 mL and increasing it up to a maximum of 2500 mL, while avoiding excessive airway pressure.

**Outcomes:**

Respiratory function was evaluated by objective measures using chest wall mobility, inspiratory capacity (IC), vital capacity (VC), tidal volume (TV), and subjective measures using the visual analogue scale (VAS). Both objective and subjective scales showed significant improvements. The patient was weaned off ventilation 48 days after the initiation of mLVR therapy.

**Lessons:**

The mLVR therapy is an effective respiratory rehabilitation for patients with weakened respiratory muscles or tracheostomy. It allows the intensity of treatment to be adjusted based on the patient’s symptoms, making it both highly effective and safe. This case suggests that mLVR therapy may be effective in treating severe, immunotherapy-resistant respiratory failure in AMA-positive myositis.

## 1. Introduction

Inflammatory myopathy is an autoimmune disease characterized by progressive muscle weakness and inflammation of skeletal muscle.^[1]^ Anti-mitochondrial antibodies (AMA) are known to be associated with inflammatory myopathy and patients with AMA-positive myositis occasionally exhibit respiratory failure.^[2]^ Some cases exhibit immunotherapy-resistant respiratory failure, which requires ventilation.^[2]^ Indeed, in a retrospective series of 18 patients with myogenic constrictive respiratory lesions, some patients required home ventilation despite immunotherapy with corticosteroids and immunosuppressive drugs, including azathioprine and cyclosporine A.^[3]^ Although such a condition induces declined activity of daily living (ADL), effective therapy has not been proven.

Lung volume recruitment (LVR) therapy, which employs a bag-valve mask (BVM), is effective for neuromuscular disorders.^[4]^ However, LVR therapy is challenging in the presence of progressive bulbar paralysis or a tracheostomy in addition to respiratory failure due to decreased respiratory muscle strength. An alternative method for continuous LVR therapy that does not require air stacking is required in such cases. For involuntary breath stacking, a medical device known as the LIC TRAINER (LT) (components from Cater Technologies, Japan) is available in Japan.^[5]^ The LT is an airtight device with a 1-way valve, safety valve, and expiratory relief valve. LVR therapy with LT has been documented to be an effective treatment for respiratory failure in amyotrophic lateral sclerosis.^[5]^ Furthermore, the previous case report on coronavirus disease-2019 has indicated the importance of prompt intervention in order to prevent respiratory muscle deterioration, particularly in cases involving immune suppression or infection.^[6]^ Herein, this case report describes a patient with AMA-positive myositis who recovered from ventilation to spontaneous breathing after the modified LVR (mLVR) therapy with LT. This case report may provide an opportunity to develop a treatment for severe immunotherapy-resistant respiratory failure in AMA-positive myositis.

## 2. Case report

**Figure 1.**
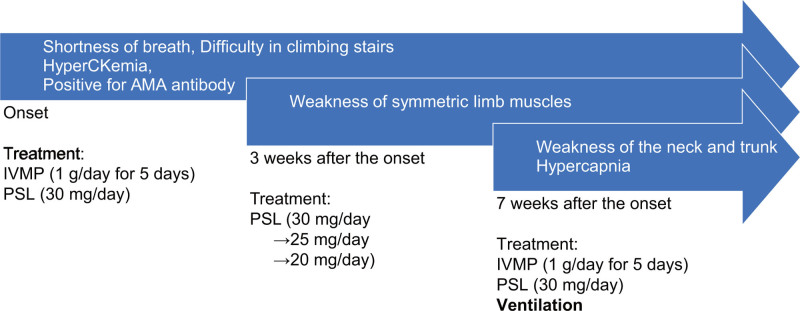
The diagnostic timeline. IVMP = intravenous methylprednisolone, PSL = prednisolone.

A 60-year-old man experienced general fatigue and shortness of breath. His condition gradually worsened, and he was admitted to our department with difficulty climbing stairs and developed severe shortness of breath approximately 4 months after onset (Fig. 1). His medical and family histories were unremarkable, with no comorbidities or relevant lifestyle factors that could have influenced disease progression. He mostly did desk work and lived with his wife and children. Upon admission, the patient had a notable degree of proximal muscle weakness. Blood test results revealed hyperCKemia (750 U/L). The serum AMA antibody was positive (187.9 U/mL; cut off: <10 U/mL). A muscle MRI revealed diffuse abnormal hyperintensities in both lower limb muscles. Needle electromyography demonstrated fibrillation and positive sharp waves at rest with early recruitment, suggesting myogenic changes. First, intravenous methylprednisolone (1 g/d) was administered for a period of 5 days, after which oral prednisolone was commenced (40 mg/d). After therapy, his muscle weakness gradually improved, but shortness of breath remained. The dosage of oral prednisolone was gradually decreased to 5 mg/wk. He was discharged when the prednisolone dosage reached 30 mg/d.

Upon reduction of the dosage to 20 mg of oral prednisolone, however, the patient exhibited a rapid and pronounced deterioration in respiratory function, requiring readmission to our department (1 month after the first steroid therapy). On the second admission, he showed the objective symmetric weakness of the proximal upper and lower extremities. The neck flexors also weaken. A muscle biopsy was not performed as the patient refused due to very strong concerns about pain. Based on the European League Against Rheumatism/American College of Rheumatology classification criteria for adult and juvenile idiopathic inflammatory myopathies,^[7]^ the age of onset ≥ 40 years (2.1 point), the progressive muscle weakness of proximal upper (0.7 point) and lower extremities (0.8 point) with proximal muscles dominance (0.9 point), the neck flexors weakness (1.9 point), difficulty in swallowing (0.7 point), and elevated serum levels of creatine kinase (1.3 point) indicated the definite idiopathic inflammatory myopathy (total: 8.4 points). Therefore, this patient was diagnosed with AMA-positive myositis. Notably, his thoracic mobility was decreased, resulting in CO_2_ narcosis (pO_2_: 75.4 mm Hg; pCO_2_: 104.7 mm Hg), requiring ventilation. Two courses of intravenous methylprednisolone (1 g/d) for 3 days followed by oral prednisolone (45 mg/d) for myopathy. However, his respiratory function remained compromised, resulting in a tracheostomy.

To address the patient’s respiratory muscle failure, we firstly considered the conventional LVR therapy. However, the conventional LVR therapy was thought to be challenging in the presence of a tracheostomy in addition to respiratory failure due to decreased respiratory muscle strength. To overcome this, we selected mLVR therapy with LT (Supplementary Video S1, Supplemental Digital Content, http://links.lww.com/MD/O171). Indeed, the benefits of integrating respiratory interventions to enhance pulmonary outcomes in patients with compromised respiratory function have been described in the previous case report.^[8]^ First, the BVM was attached to the primary port of the LT. The physiotherapist adjusted the expiratory valve of LT to avoid excessive airway pressure. The physiotherapist slowly applied pressure several times using the BVM while checking the airway pressure.

**Figure 2.**
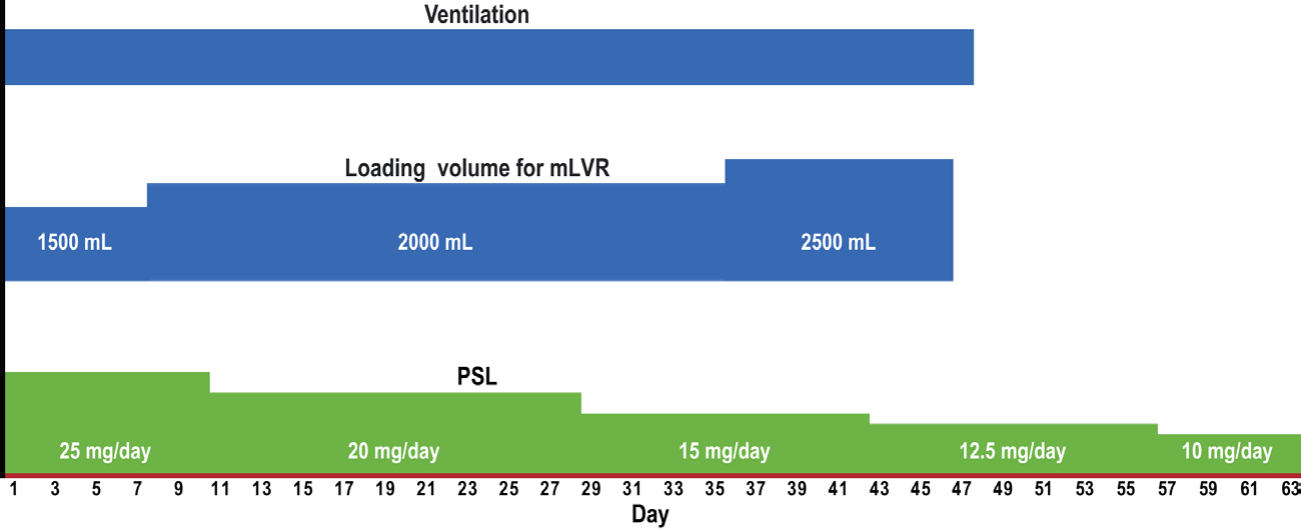
The clinical course after modified lung volume recruitment therapy. The timing of each therapy is shown. Day 1 is the day of the initiation of mLVR therapy. mLVR = modified lung volume recruitment, PSL = prednisolone, mLVR = modified lung volume recruitment.

The bagging started with 3 cycles of 500 mL each, adjusted to the patient’s breathing pattern. The lungs and alveoli were sufficiently inflated with 1500 mL of inspired air and maintained for at least 5 seconds. This was repeated 5 times to complete a set, and a total of 2 sets were performed at least twice a day. For step-up, the resistance felt during bagging and the airway pressure gauge on the device were checked to ensure that the pressure remained below 40 cmH_2_O. Patient comfort, as indicated by discomfort or facial expression, was used as a guide and the number of bagging cycles was gradually increased in steps: 4 cycles (2000 mL) in 1 set and then 5 cycles (2500 mL) (Fig. 2). LT is a forced inspiration device, and while additional inspiration is provided through a bag-valve above the patient’s maximum inhalation, some temporary discomfort was observed. However, there was no need to stop the LT or reduce the tidal volume (TV).

This report assessed the patient’s respiratory conditions by the following factors (Table 1): chest wall mobility associated not only with the respiratory muscle strength but also with the lung and chest wall compliance, medical research council (MRC) score for limb muscle strength, vital capacity (VC), inspiratory capacity (IC), TV for objective respiratory scale, and visual analogue scale (VAS) for subjective respiratory scale. The chest wall mobility was evaluated by measuring the difference in thoracic circumference between maximal and maximal expirations at the third intercostal space, xiphoid process, and level of the 10th intercostal space.^[9]^ The VC was evaluated by averaging 3 measurements obtained from a tracheal cannula using a respirometer (HALOSCALE) under spontaneous breathing. IC was assessed by the average of 3 measured values during deep breathing using a ventilator (PEEP: 5). TV was assessed by averaging the 3 measured values obtained when the patient was breathing at rest under ventilatory conditions (PEEP: 5).

**Table 1:** Evaluation of respiratory muscles and respiratory function.

		Day 8	Day 11	Day 14	Day 37
MRC		49	49	50	53
Difference in thoracic circumference (mm)	Third intercostal space	0.2	0.4	1	2.1
	Xiphoid process	0.4	0.6	1.2	2.8
	10th intercostal space	0.2	0.2	2	4
VC (mL)	Pre-mLVR	1063.3	1112.5	1155.0	1502.5
	Post-mLVR	1193.3	1170.0	1240.0	1462.5
	Difference (Post-Pre)	130.0	57.5	85.0	−40.0
IC (mL)	Pre-mLVR	776.0	798.7	1052.0	1241.0
	Post-mLVR	1081.0	999.3	1253.3	1293.3
	Difference (Post-Pre)	305.0	200.7	201.3	52.3
TV (mL)	Pre-mLVR	121.7	128.0	102.7	300.0
	Post-mLVR	137.3	185.7	107.7	302.0
	Difference (Post-Pre)	15.7	57.7	5.0	2.0
VAS (mm)	Pre-mLVR	78	64	48	32
	Post-mLVR	70	44	43	32
	Difference (Post-Pre)	8	20	5	0

Abbreviations: IC = inspiratory capacity, mLVR = modified lung volume recruitment, MRC = medical research council, TV = tidal volume, VAS = visual analogue scale, VC = vital capacity. Note that not only objective but also subjective respiratory scales showed improvement after mLVR therapy.

Chest wall mobility was significantly improved, such that the difference in thoracic circumference between maximum inspiration and expiration was increased (Table 1). The improvement in the MRC scale scores was weaker than that in the respiratory-associated scales. The objective and subjective respiratory scales also improved with mLVR therapy. The difference between pre- and post-mLVR therapies VAS scores exhibited patterns comparable to those observed for TV. We compared the respiratory function parameters measured in this patient with predicted normal values. Predicted normal values were calculated based on the patient’s height (155 cm) and age (60 years) or were obtained from previous literature. For TV, it was assumed that predicted normal values would not be applicable because the measurement was taken from the tracheal cannula. To calculate the minimum TV under ventilation, we used the predicted body weight from large-scale ARDS RCTs, calculated as predicted body weight × 6 mL/kg.^[10]^ The predicted body weight for male patients is calculated as 50 + 0.91 × (height cm − 152.4), resulting in a TV of 314 mL for this patient. The predicted normal VC was calculated using the Baldwin formula.^[11]^ The VC of male patients was calculated as (27.63 − 0.112 × age) × height in centimeters, resulting in 3240 mL. According to the previous study,^[12]^ the predicted mean difference in chest circumference at the xiphoid process for individuals aged 50 to 75 years was 4.7 cm with a standard deviation of 1.4 cm. When we compared these data with those of this patient, TV had improved to a value close to the predicted normal value, whereas the difference in chest circumference was smaller than average, and VC improved to only about half the predicted normal value. Importantly, there was no need for reintubation after extubation and the patient did not experience any respiratory distress. The patient was successfully weaned from ventilation 48 days after the initiation of mLVR therapy. Nasogastric feeding was provided during mechanical ventilation, but early swallowing training was initiated after extubation. This resulted in early improvement in swallowing function. The patient required temporary swallow training foods, but was able to start regular meals after 56 days and was discharged 63 days after the initiation of mLVR therapy.

## 3. Discussion

Our patient presented with severe immunotherapy-resistant respiratory failure due to AMA-positive myositis that required ventilation and tracheostomy. His chronic respiratory failure significantly improved with mLVR therapy (Table 1). In addition, after being weaned from the ventilator with mLVR therapy, the patient became more mentally positive, actively participated in rehabilitation, and began eating a high-protein diet to strengthen his muscles. Conventional LVR therapy, it was difficult to perform in patients with tracheostomy or respiratory muscle paralysis because they were unable to hold their breath. However, by using LT, passive breath-holding can be achieved. Additionally, increasing the pressure duration leads to improvements in lung compliance. Furthermore, the ability to adjust ventilation volume and pressure duration based on the patient’s discomfort or complaints enhances safety. In a previous case series study,^[2]^ 70% of patients with AMA-positive myositis exhibited a VC of 80% or less, and a considerable proportion of patients developed respiratory failure. In some patients, respiratory failure became severe, necessitating the use of noninvasive positive pressure ventilation or invasive positive pressure ventilation, which led to respiratory management even in the chronic phase. Our case suggests that mLVR therapy may contribute to the treatment of severe respiratory failure.

Patients with neuromuscular disorders experience a progressive decrease in VC and an increase in work of breathing as a result of progressive inspiratory muscle weakness and an augmenting elastic load induced by a reduction in lung and thoracic compliance.^[13]^ It has been shown that maximal insufflation is of significant importance in improving respiratory function in these patients with a VC of <1500 mL.^[14]^ However, LVR is known to cause musculoskeletal discomfort and side effects such as unexplained pain, dizziness, shortness of breath, and anxiety.^[15]^ Moreover, a randomized controlled trial showed no significant improvement in VC after LVR in patients with neuromuscular diseases such as amyotrophic lateral sclerosis or muscular dystrophy.^[15]^ In our case, the efficacy of mLVR therapy was observed after therapy and was sustained, with eventual recovery from ventilation to spontaneous breathing. The patient reported only transient discomfort during mLVR therapy, and no discontinuation of respiratory rehabilitation was required as a result. As seen in the VAS, the subjective respiratory scale also improved after therapy. Initiation with a loading volume of 1500 mL and the use of an expiratory relief valve may have contributed to the prevention of complications due to treatments such as pneumothorax, maintenance of the patient’s mental status, and improvement of respiratory failure. Moreover, LVR may be effective in improving respiration due to inflammatory myositis when used in addition to immunotherapy.

It is imperative to note that this report represents a single case study. The lack of a control or comparison group for this report makes it difficult to draw conclusions about the effectiveness of mLVR therapy. Further research, including not only experimental studies but also larger patient cohorts, is essential to develop effective therapeutic strategies to manage respiratory failure due to AMA-positive myositis. In addition, we were unable to discuss the importance of neuromodulation techniques such as transcutaneous neuromodulation^[16]^ or adjunctive electrotherapy for respiratory muscle rehabilitation^[17]^ those are known to effective to improve respiratory functions, because we only provided mLVR therapy. The combination of mLVR therapy with these neuromodulation techniques may result in an enhanced therapeutic efficacy.

Nonetheless, our findings convey an important message to clinicians managing cases of chronic respiratory failure due to AMA-positive myositis. The severity of respiratory failure in these patients was associated with ADL.^[2]^ Our case showed improved not only objective but also subjective outcomes, that is, the VAS scale (Table 1). The VAS is known to have good validity as an indicator of ADL.^[18]^ Detecting respiratory failure as early as possible and initiating respiratory rehabilitation at the right time would be beneficial in maintaining ADLs in patients with AMA-positive myositis.

## Acknowledgments

We thank the staff at Showa University Fujigaoka Hospital and Showa University Fujigaoka Rehabilitation Hospital for their effort in rehabilitation and Tomoko Nagami for administrative assistance.

## Author contributions

**Conceptualization:** Seiya Takahashi, Ryuta Kinno.

**Data curation:** Seiya Takahashi.

**Funding acquisition:** Ryuta Kinno.

**Resources:** Seiya Takahashi, Hiroyasu Inoue, Rihito Mitsuhashi, Kazuki Komaba, Akinori Futamura, Satoshi Nogawa.

**Supervision:** Ryuta Kinno.

**Validation:** Seiya Takahashi, Ryuta Kinno.

**Visualization:** Seiya Takahashi, Ryuta Kinno.

**Writing – original draft:** Seiya Takahashi, Ryuta Kinno.

**Writing – review & editing:** Seiya Takahashi, Hiroyasu Inoue, Shizuki Amano, Takahiro Shinohara, Sara Mori, Kaho Onizawa, Yoko Nabeshima, Hiroyasu Komuro, Taro Yasumoto, Rihito Mitsuhashi, Daishi Watanabe, Kazuki Komaba, Akinori Futamura, Satoshi Nogawa, Ryuta Kinno.

## Supplementary Material


